# Measurement of Available Carbohydrates in Cereal and Cereal Products, Dairy Products, Vegetables, Fruit, and Related Food Products and Animal Feeds: First Action 2020.07

**DOI:** 10.1093/jaoacint/qsab019

**Published:** 2021-02-05

**Authors:** Barry V McCleary, Ciara McLoughlin

**Affiliations:** 1 Megazyme, Bray Business Park, Southern Cross Road, Bray, County Wicklow, Ireland; 2 MGZ Consultants, Murrumburrah, Eden Road, Greystones, Wicklow, Ireland

## Abstract

**Background:**

The level of available carbohydrates in our diet is directly linked to two major diseases: obesity and Type II diabetes. Despite this, to date there is no method available to allow direct and accurate measurement of available carbohydrates in human and animal foods.

**Objective:**

The aim of this research was to develop a method that would allow simple and accurate measurement of available carbohydrates, defined as non-resistant starch, maltodextrins, maltose, isomaltose, sucrose, lactose, glucose, fructose, and galactose.

**Method:**

Non-resistant (digestible) starch is hydrolyzed to glucose and maltose by pancreatic α-amylase (PAA) and amyloglucosidase at pH 6.0 with shaking or stirring at 37°C for 4 h. Sucrose, lactose, maltose, and isomaltose are completely hydrolyzed by specific enzymes to their constituent monosaccharides, which are then measured using pure enzymes in a single reaction cuvette.

**Results:**

A method has been developed that allows the accurate measurement of available carbohydrates in all cereal, vegetable, fruit, food, and feed products, including dairy products.

**Conclusions:**

A single-laboratory validation was performed on a wide range of food and feed products. The inter-day repeatability (RSD_r_, %) was <3.58% (w/w) across a range of samples containing 44.1–88.9% available carbohydrates. The LOD and LOQ obtained were 0.054% (w/w) and 0.179% (w/w), respectively. The method is all inclusive, specific, robust, and simple to use.

**Highlights:**

A unique method has been developed for the direct measurement of available carbohydrates, entailing separate measurement of glucose, fructose, and galactose, information of value in determining the glycemic index of foods.

##  

Traditionally, “available carbohydrates,” i.e., carbohydrates available for digestion in the human small intestine, have been determined by either direct or indirect measurement. In 1929, McCance and Lawrence (1) introduced the direct analysis of D-glucose, D-fructose, sucrose, lactose, maltose, and starch (including maltodextrins) in foods and then summed these to obtain a total value for “available carbohydrates.” This has been the basis of carbohydrate analysis in the United Kingdom since that time, as detailed by Southgate (2). In the United States, the “by difference” method for determining total carbohydrates was introduced by Atwater and Woods in 1896 (3) and is still in use today. The moisture, protein, fat, and ash content of a food are determined and then subtracted from the total weight of the food and the remainder, or “difference,” is considered to be total carbohydrate. More recently, “net carbohydrates” has been determined by subtracting dietary fiber from the total carbohydrates value. This value is meant to equate with “available carbohydrates” determined directly, but, the “by difference” figure includes non-carbohydrate components such as lignin, organic acids, tannins, waxes, and some Maillard products. Also, it combines all the analytical errors from the other analyses (4). To date, methods for the direct measurement of available carbohydrates have been difficult to implement and are non-specific, as detailed by Southgate (2).

In the measurement of available carbohydrates, total starch has traditionally been measured instead of just digestible starch, and sucrose hydrolysis was achieved with invertase (β-fructofuranosidase). Englyst et al. (5) developed methodology for the measurement of digestible starch and resistant starch and this methodology is widely used and cited. These authors considered that digestion of starch by pancreatic α-amylase (PAA) plus amyloglucosidase (AMG) was complete within 2 h of incubation at 37°C. However, since literature indicate that the average time of residence of food in the human small intestine is ∼ 4 h (6, 7), we have settled on this incubation time in developing the current methodology. Starch that is digested during this time period is part of the carbohydrate that is absorbed in the human small intestine. Traditionally, the measurement of sucrose has involved hydrolysis to glucose and fructose by invertase, with subsequent measurement of the glucose and fructose. However, invertase also hydrolyzes the lower degree of polymerization (DP) fructo-oligosaccharides (FOS), resulting in overestimation of sucrose in those samples that contain FOS. In the current study, maltose is hydrolyzed to glucose by maltase, and sucrose is specifically hydrolyzed to glucose and fructose by a sucrase enzyme that has no action on FOS (7, 8). Lactose is hydrolyzed to glucose and galactose by a specific β-galactosidase (MZ104). This β-galactosidase also has some action on GOS, if present in the sample, leading to an overestimation of available carbohydrates for such samples. Isomalto-oligosaccharides (IMO) find some application in food preparations as a digestion-resistant carbohydrate. However, in the presence of the PAA/AMG used in this method, IMO are hydrolyzed mainly to glucose and isomaltose, an oligosaccharide that is digested in the human small intestine (7, 9, 10). Consequently, in the current procedure, isomaltose is hydrolyzed to glucose using oligo-1,6-α-glucosidase. Galactose, glucose, and fructose are measured employing high-purity and specific enzymes.

### AOAC Official Method 2020. 07 Available Carbohydrates in Cereal and Cereal Products, Dairy Products, Vegetables, Fruit and Food Products, and Animal Feeds First Action 2020

This method is an extension of AOAC Method **2017.16** (7, 8) for the measurement of total dietary fiber. Incubation conditions with PAA/AMG are the same, however scaled down two-fold. Aliquots of the hydrolysate are incubated with sucrase, maltase, and β-galactosidase and total released galactose, glucose, and fructose are measured. The method is commercially available as the Available Carbohydrates Assay Kit (Megazyme Cat. No. K-AVCHO).

#### A. Method

**(a)** *Scope of the method.—*

*(1)* *Target analyte.—*Digestible starch, maltodextrins, maltose, IMO, isomaltose, sucrose, lactose, glucose, fructose, and galactose. The specific β-galactosidase (MZ104) employed in this method to hydrolyze lactose also has some action on specific GOS and thus could lead to an overestimation of available carbohydrates in samples containing GOS.

*(2)* *Matrices.—*For this validation, the Available Carbohydrates Assay Kit (K-AVCHO) was tested with multiple matrixes.

  *(a)* *Cereal grains, breakfast cereals, bread, biscuits, and pasta products.—*Conventional barley flour (Lot No. 60301; Megazyme, Bray, Ireland), Barley MAX**^®^** (The Healthy Grain Pty. Ltd, South Yarra, Australia), Kellogg^**^®^**^ breakfast cereals (cornflakes, All Bran^**^®^**^, Special K^**^®^**^, Frosties^**^®^**^), Weetabix^**^®^**^ (Weetabix Limited UK), Brennan’s whole meal bread, cracker biscuits, other biscuits, and Roma^**^®^**^ Maceroni pasta were from Tesco Ireland (Greystones, County Wicklow, Ireland).

  *(b)* *Dairy products.—*Cow & Gate infant formula, and various drink powders; Cadbury dairy milk chocolate were obtained from Tesco Ireland.

  *(c)* *Commercial starches and other polysaccharides.—*Corn starch (regular maize starch; cat. no. S-4126), high amylopectin corn starch (Cat. no. S-9679), potato starch (Cat. No. S-4251), wheat starch (Cat. No. S-5127), rice starch (Cat. No. S-7260), and potato amylose (Cat. No. A-9262) were from Sigma–Aldrich Ireland Ltd (Dublin, Ireland). Hylon VII^®^ (Ref. 98GH8401) was from National Starch and Chemical Co. (Bridgewater, CT; now Ingredion). Fibersol 2^**^®^**^ was from Matsutani Chemical Co. (Hyogo, Japan). Fibersym^**^®^**^ was from MGP ingredients (Atchison, Kansas). Raftaline^**^®^**^ (Orafti GR^**^®^**^) Native chicory fructan and Raftilose^**^®^**^ (Orafti P95^**^®^**^) hydrolyzed fructan were obtained from BENEO Tienen (Tienen, Belgium).

  *(d)* *Vegetables and fruit.—*Fresh vegetables and fruit and canned beans used in this study were from Tesco Ireland.

  *(e)* *Animal feeds.—*Connolly’s Red Mills Layer^**^®^**^ hen pellets were obtained from Connolly’s Red Mills (Goresbridge, Co. Kilkenny, Ireland). Gain^**^®^**^ Soybean meal, Gain Freedom^**^®^**^ mix low starch horse feed, Gain^**^®^**^ Drive dairy feed complete, Gain**^®^**small dog adult kibble, Gain feeds flaked maize, and Gain^**^®^**^ Premium Cuts wet dog food were obtained from Gain Animal Feeds (Portlaoise, Ireland). Brett Brothers swine feed was from Glanbia, Ireland and Purina GoCat^**^®^**^ dry cat food was from Tesco Ireland.

  *(f)* *Canned beans and peas.—*Commercial canned beans and peas (cooked, boiled, with salt) were from Tesco Ireland.

#### B. Principle

Test portions to be analyzed are suspended in maleate buffer (pH 6.0) containing PAA and AMG and continually mixed or stirred at 37°C for 4 h. During this time, digestible starch is hydrolyzed to glucose plus traces of remaining maltose, as shown in Equation 1.
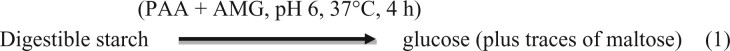


Test solutions are then removed, diluted, and centrifuged. Aliquots of this diluted solution are incubated with a mixture of sucrase, maltase, and β-galactosidase at pH 6.5 and 30°C for 30 min, during which time sucrose is specifically hydrolyzed to glucose and fructose by sucrase enzyme, maltose is hydrolyzed to glucose, and lactose is hydrolyzed to galactose and glucose by β-galactosidase (Equations 2–4).
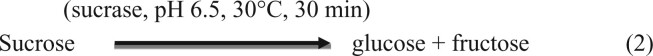



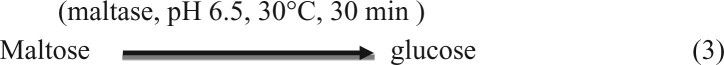



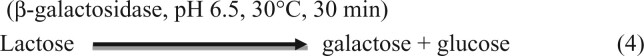


If isomaltose or isomalto-oligosaccharides are present in the sample, an extra enzyme, oligo-1,6-α-glucosidase (Megazyme Cat. No. O-AGUM), is added to the above incubation mixture (Equation 5).
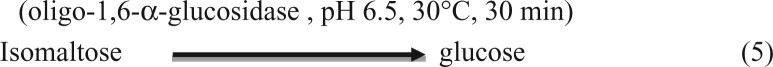


In the presence of the enzymes galactose dehydrogenase (GalDH) and galactose mutarotase (GalM), β-D-galactose is oxidized by nicotinamide-adenine dinucleotide phosphate (NADP^+^) to D-galactonate with the formation of reduced nicotinamide-adenine dinucleotide phosphate (NADPH) (Equation 6).
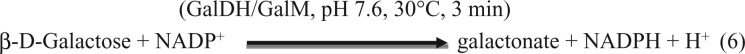


The amount of NADPH formed in this reaction is stoichiometric with the amount of D-galactose. It is the NADPH which is measured by the increase in absorbance at 340 nm.

Glucose and fructose are phosphorylated by the enzyme hexokinase (HK) and adenosine-5’-triphosphate (ATP) to glucose-6-phosphate (G-6-P) and fructose-6-phosphate (F-6-P) with the simultaneous formation of adenosine-5’-diphosphate (ADP) (Equations 7 and 8).
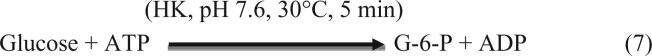



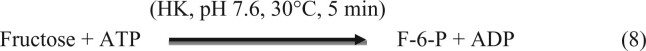


In the presence of the enzyme glucose-6-phosphate dehydrogenase (G6P-DH), G-6-P is oxidized by NADP^+^ to gluconate-6-phosphate with the formation of reduced NADPH (Equation 9).
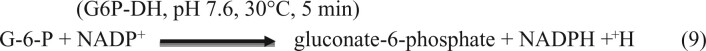


The amount of NADPH formed in this reaction is stoichiometric with the amount of glucose. It is the NADPH which is measured by the increase in absorbance at 340 nm.

On completion of reaction 9, F-6-P is converted to G-6-P by phosphoglucose isomerase (PGI) (Equation 10).
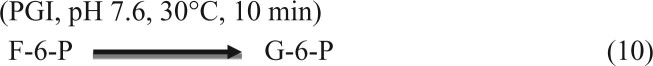


The G-6-P formed reacts in turn with NADP^+^ forming gluconate-6-phosphate and NADPH, leading to a further rise in absorbance that is stoichiometric with the amount of fructose.

The method is simple to use and the absorbance response for galactose, glucose, and fructose is the same (Figure **2020.07A**).

**Figure 2020.07A.**
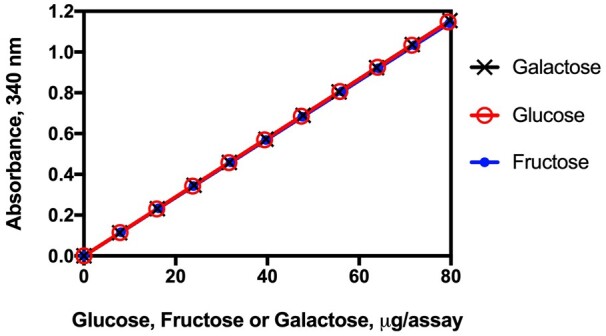
Linearity of the absorbance response in the enzymatic reactions in the measurement of galactose, glucose, and fructose. r^2^ = 0.9999 for each sugar.

#### C. Apparatus

- *Grinding mill.—*Centrifugal, with a 12-tooth rotor and 0.5 mm sieve, or similar device. Alternatively, a cyclone mill can be used for small test laboratory samples provided they have sufficient air flow or other cooling to avoid overheating samples.
- *Meat mincer.*—Hand-operated or electric, fitted with a 4.5 mm screen.
- *Water bath.*—To accommodate a submersible magnetic stirrer (e.g., 2mag Mixdrive 15^®^) with an immersion heater (e.g., Julabo^®^ Immersion Circulator, Julabo, Seelbach, Germany) (Figure **2020.07B**).
- *Submersible magnetic stirrer.*—(e.g., 2mag Mixdrive 15 submersible magnetic stirrer (Munich, Germany).
- *Water bath*.—Linear motion, shake speed 200 strokes/min, 35 mm stroke length, capable of maintaining temperature of 37± 1°C. With clips or springs to allow attachment of tubes. http://www.keison.co.uk/grantinstruments_ols200.shtml (Figure **2020.07C**). Shaking speed must be sufficient to keep the sample completely suspended during the incubation period.
- *Spectrophotometer.*—Capable of operating at 340 nm, (10 mm path length), e.g., MegaQuant^TM^ Wave Spectrophotometer (Megazyme Cat. No. D-MQWAVE).
- *Analytical balance.*—0.1 mg readability, accuracy, and precision.
- *Freeze-drier.*—e.g., Virtis Genesis^®^ 25XL or similar (Biopharma Process Systems, Biopharma House, Winchester, UK).
- *Microfuge centrifuge.*—Capable of 13 000 rpm (∼15 500 *g*).
- *Disposable 2.0 mL polypropylene microfuge tubes*.—e.g., Sarstedt Cat. No. 72.691. Sarstedt Ltd (Drinagh, Co Wexford, Ireland).
- *pH meter.*—e.g., Seven Easy pH Mettler Toledo.
- *Vortex mixer.*—e.g., Daihan Scientific VM10.
- *Moisture analyzer.*—e.g., OHAUS MB45.
- *Fat extraction system.*—e.g., ANKOM XT15 Extractor.
- *Magnetic stirrer.*—e.g., IKA KMO 2 basic stirrer.
- *Magnetic stirring bars.*—e.g., Fisherbrand^TM^ PTFE Stir Bars 20 × 6 mm ridged.
- *Digestion bottles.*—250 mL, soda-lime glass, wide-mouth bottles with polyvinyl lined cap (e.g., Fisherbrand^®^ Cat. No. FB73219).
- *Laboratory timer.*
- *Micro-pipettors.*—e.g., Gilson Pipetman^®^ (100 and 20 µL) used for dispensing 100 µL of test solutions and 20 µL of enzyme preparations (Woodside Industrial Estate, Dunstable, United Kingdom).
- *Positive displacement pipettor.*—e.g., HandiStep^®^ with 5 mL tip to dispense 0.5 mL of 95% v/v ethanol and to dispense 0.1 mL sucrase/β-galatosidase, 0.1 mL Imidazole buffer and 0.1 mL NADP^+^ solution; HandiStep with 25 mL tip to dispense 2.5 mL aliquots of PAA/AMG; HandiStep with 12.5 mL tip to remove 1.0 mL aliquots from incubation solutions.
- *Dispensers.*—e.g., Brand HandyStep dispensette S Digital 2.5–25 mL Cat. No. 4600351 to dispense 17.5 mL of sodium maleate buffer **[D(m)]** and 25 mL of distilled water.
- *Polypropylene tubes.*—Sarstedt polypropylene tube; 40 mL, 30 × 84 mm (Cat. No. 62.555) or equivalent.
- *Polypropylene sheet with precision cut holes.*—To hold and align 40 mL polypropylene tubes on the stirrer plate of the 2mag Mixdrive 15 submersible magnetic stirrer (Figure **2020.07B**).

**Figure 2020.07B.**
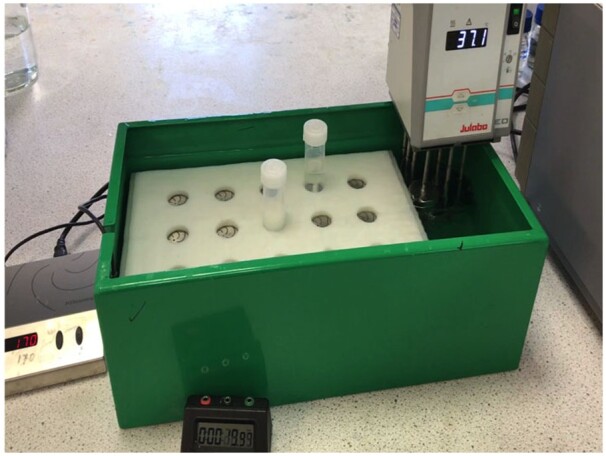
Designed water bath (Cat. No. D-TDFBTH) with submersible 2mag Mixdrive 15 submersible magnetic stirrer and a designed polypropylene tube holder (Megazyme Cat. No. D-PPTH) **[C(v)**]. Samples (∼0.5 g) incubated with enzyme mixture in 40 mL polypropylene tubes with stirrer bar.

#### D. Reagents

- *EtOH (95% v/v).*
- *Imidazole buffer (2 M, pH 7.6) containing magnesium chloride (100 mM) and sodium azide (0.02% w/v) as a preservative.—*Stable for > 2 years at 4°C.
- *NADP^+^ (0.84 mM) plus ATP (2.5 mM).—*Stable for > 5 years stored dry below –10°C.
- *Stock PAA plus AMG powder.—*PAA (40 KU/g) plus AMG (17 KU/g) as a freeze-dried powder mixture. *Note*: One unit of AMG activity is the amount of enzyme required to release one µmole of d-glucose from soluble starch per minute at 40°C and pH 4.5; one unit of PAA activity is the amount of enzyme required to release one µmole of *p*-nitrophenyl from Ceralpha reagent per min at 40°C and pH 6.9; AOAC Method **2002.01**). PAA/AMG preparations should be essentially devoid of β-glucanase, β-xylanase and detectable levels of free d-glucose. Stable for > 4 years at −20°C.
- *PAA (0.8 KU/mL)/AMG (0.34 KU/mL).—*
*(1)* Immediately before use, dissolve 0.5 g of PAA/AMG powder **[D(d)]** in 25 mL of sodium maleate buffer (50 mM, pH 6.0 plus 2 mM CaCl_2_) **[D(m)]** and stir for approx. 5 min. Store on ice during use. Use on the day of preparation*.*
*(2)* Alternatively, some individuals are allergic to powdered PAA and/or AMG. In this instance, engage an analyst who is not allergic to prepare the powdered enzymes as an ammonium sulphate suspension as follows: gradually add 2.5 g of PAA/AMG powder mix (PAA 40 KU/g plus AMG 17 KU/g) to 35 mL sodium maleate buffer (50 mM, pH 6.0 plus 2 mM CaCl_2_) **[D(m)]** in a 100 mL beaker on a magnetic stirrer in a laboratory hood and stir until the enzymes are completely dissolved (∼5 min). Add 17 g of granular ammonium sulphate and dissolve by stirring. Adjust the volume to 50 mL with ammonium sulphate solution **[D(o)]** and store at 4°C. (This preparation **[D(e)*(***2*)***]** contains PAA at 2 KU/mL and AMG at 0.85 KU/mL). Stable at 4°C for 3 months].
- *Sucrase (270 U on sucrose) plus β-galactosidase (1000 U on p-nitrophenyl β-D-galactoside).—*Freeze-dried powder. Stable for >3 years stored dry below –10°C. Before use, dissolve the enzyme mixture in 10.5 mL of 50 mM sodium maleate buffer (pH 6.5) containing BSA **[D(n)],** divide into aliquots of ∼ 4 mL and store in polypropylene tubes at <–10°C between use.
- *Oligo-1,6-α-glucosidase (1000 U/mL).—*Suspension in 3.2 M ammonium sulphate plus sodium azide (0.02% w/v). Stable for >2 years at 4°C.
- *Galactose dehydrogenase (200 U/mL) plus galactose mutarotase suspension (4.1 mg/mL*)*.—*Suspension in 3.2 M ammonium sulphate plus sodium azide (0.02%, w/v). Stable for >2 years at 4°C.
- *Hexokinase (425 U/mL) plus glucose-6-phosphate dehydrogenase (110 U/mL).—*Suspension in 3.2 M ammonium sulphate plus sodium azide (0.02% w/v). Stable for >2 years at 4°C.
- *Phosphoglucose isomerase suspension (1000 U/mL).—*Suspension in 3.2 M ammonium sulphate plus sodium azide (0.02% w/v). Stable for >2 years at 4°C.
- *D-Glucose, D-fructose plus D-galactose standard solution (5 mL, 0.2 mg/mL of each sugar in 0.02% w/v sodium azide solution*)*.—*Stable for >2 years at 4°C.
- *Available carbohydrates control (∼10 g).—*Available carbohydrates value shown on the label. Stable for >2 years at 4°C.
- *Sodium maleate buffer (50 mM, pH 6.0) plus CaCl_2_ (2 mM).*—Dissolve 11.6 g of maleic acid in 1600 mL of deionized water and adjust the pH to 6.0 with 4 M (160 g/L) NaOH solution. Add 0.6 g of calcium chloride dihydrate (CaCl_2_·2H_2_O); dissolve and adjust the volume to 2 L. Store in a well-sealed bottle (e.g., Duran^®^) and add two drops of toluene to prevent microbial infection. Stable for ∼ 1 year at 4°C.
- *Sodium maleate buffer (50 mM, pH 6.5) containing BSA (0.5 mg/mL), and sodium azide (0.02% w/v).*—Dissolve 5.8 g of maleic acid in 800 mL of deionized water and adjust the pH to 6.5 with 4 M (160 g/L) NaOH solution. Add 0.5 g of BSA and 0.2 g of sodium azide and dissolve by stirring. Adjust the volume to 1 L. Store in a well-sealed bottle (e.g., Duran). Stable for ∼1 year at 4°C.
- *Ammonium sulphate solution, 50% w/v.*—Add 50 g of ammonium sulphate to 75 mL of distilled water and dissolve by stirring. Adjust volume to 100 mL with distilled water. Store in a well-sealed bottle (e.g., Duran). Stable for >2 years at room temperature.

Items **(b)–(f)** and **(h)–(l)** are supplied in the Available Carbohydrates kit (K-AVCHO) available from Megazyme (Bray, County Wicklow, Ireland). Oligo-1,6-α-Glucosidase (Cat. No. E-OAGUM) is also available from Megazyme. Current preparations of sucrase plus β-galactosidase [**D(f)**] also contain oligo-1,6-α-glucosidase [**D(g)**]. Preparations of reagents and buffers which meet the criteria as specified in the method above may also be used.

#### E. Safety Considerations

The general safety measures that apply to all chemical substances should be adhered to. For more information regarding the safe use and handling of the Available Carbohydrates Assay Kit reagents (K-AVCHO) refer to the K-AVCHO SDS document that is downloadable from where the product appears on the Megazyme website (www.megazyme.com). Some individuals are allergic to powdered PAA and/or amyloglucosidase. This enzyme preparation should be weighed and dissolved in a well-ventilated fume cupboard. The preparation can be stabilized with ammonium sulphate to reduce handling of the powder product.

#### F. Preparation of Test Materials

Collect and prepare food materials as “intended to be eaten,” i.e., cook pasta and potatoes. For dry foods, animal feeds, and breakfast cereals, grind approximately 50 g material in a grinding mill to pass a 0.5 mm sieve. Transfer all materials into wide-mouthed plastic jars, and seal and mix well by shaking and inversion. Freeze dry high-moisture- (>25% w/w) containing materials. Pour canned beans and vegetables onto a strainer and wash with demineralized water, freeze dry, and mill to pass a 0.5 mm screen. Homogenize dried fruit materials in a high-speed blender (e.g., Nutri-Bullet) and then dry the paste in a forced air oven at 40°C overnight. Further grind materials to be analyzed using a mortar and pestle. Homogenize high-fat-containing materials such as malted milk biscuits, chocolate digestive biscuits, jam and cream biscuits, shortbread finger biscuits, and Cadbury^**^®^**^ dairy milk chocolate using a high-speed blender (e.g., Nutri-Bullet). In a well-ventilated fume cupboard, transfer a portion of the homogenized material (approximately 10 g, weighed accurately) to a pre-weighed 200 mL beaker and add 50 mL of petroleum ether. Stir the mixture with a spatula for 20 s and allow the solids to settle. Carefully decant the supernatant and then repeat this process a further two times. Allow the solids in the beaker to dry in a well-ventilated fume hood and weigh. Calculate fat content. Store materials in the presence of a desiccant.

#### G. Measurement of Enzyme Activities

Measure the activity of α-amylase activity in PAA using the Ceralpha^**^®^**^ assay procedure employing non-reducing end-blocked *p*-nitrophenyl maltoheptaoside in the presence of excess levels of thermostable α-glucosidase. Perform incubations in sodium maleate buffer at pH 6.9 and 40°C as described in the α-Amylase Assay Kit (Ceralpha^®^ Method) booklet (Megazyme Cat. No. K-CERA; AOAC Official Method **2002.01**) (11). One unit of enzyme activity is defined as the amount of enzyme that releases one µmole of *p*-nitrophenol per minute under the defined assay procedure. The α-amylase activity reported is that measured at the optimal pH of 6.9. However, incubations for the measurement of digestible starch, resistant starch, and available carbohydrates were performed at pH 6.0. α-Amylase activity at pH 6.0 is ∼77% of that at pH 6.9 (12). AMG was assayed by incubating 0.2 mL of suitably diluted enzyme in 100 mM sodium acetate buffer (pH 4.5) with 0.5 mL of soluble starch (10 mg/mL) in 100 mM sodium acetate buffer (pH 4.5) at 40°C. At various time intervals, reaction tubes were heated to ∼100°C in a boiling water bath to terminate the reaction and released glucose was measured using GOPOD reagent [Glucose Assay Kit (GOPOD Format); Megazyme Cat. No. K-GLUC]. One unit of AMG is defined as the amount of enzyme required to release one µmole of D-glucose per minute at pH 4.5 and 40°C. When in admixture with PAA, AMG was assayed using AMG Assay Reagent (Megazyme Cat. No. R-AMGR3) and units of activity on starch were calculated using a conversion factor. The AMG activity reported is that measured at the optimal pH of 4.5. However, incubations for the measurement of digestible starch, resistant starch, and available carbohydrates were performed at pH 6.0. AMG activity at pH 6.0 is ∼60% of that at pH 4.5.

#### H. Assay Procedure

- *Hydrolysis of digestible starch.—*
*(1)* Weigh 0.500 ± 0.005 g test portion accurately into 30 × 84 mm (40 mL) polypropylene tube **[C(v)].**
*(2)* Wet the material with 0.5 mL of ethanol (95% v/v) **[D(a)],** add 17.5 mL of 50 mM sodium maleate buffer, pH 6.0 **[D(m)]** using a positive displacement dispenser **[C(u)]**, and stir the tube for a few minutes to ensure all material is suspended. Add 2.5 mL of PAA/AMG solution **[D(e)***(*1*)***]**, cap the tube, and incubate the reaction solution with stirring at 170 rpm on a submersible magnetic stirrer **[C(d)]** at 37°C for 4 h (Figure **2020.07B**) using a precision-cut polypropylene sheet **[C(w)]** to hold the tubes in place. *Note*: If using an (NH_4_)_2_SO_4_ suspension of this enzyme preparation **[D(e)***(*2*)***],** add 19 mL of 50 mM sodium maleate buffer **[D(m)]** to the material being analyzed followed by 1 mL of enzyme suspension. Alternatively, place the tubes horizontally (in the line of direction of shaking) in a shaking water bath **[C(e)]** (Figure **2020.07C**) set at 200 strokes/min and incubate at 37°C for 4 h.
*(3)* After 4 h, accurately transfer 1.0 mL of the reaction solution to 25 mL of cold water in a 40 mL polypropylene tube **[C(v)].** Cap the tube, mix the contents thoroughly and store at 4°C awaiting analysis. For materials containing <10% available carbohydrates content, accurately transfer 1.0 mL of the reaction solution to 5 mL of cold water and mix well.
- *Measurement of available carbohydrates.—*
*(1)* Transfer 2 mL of the above solution to a 2.0 mL polypropylene microfuge tube **[C(j)]** and centrifuge at 13 000 rpm (15 500 *g*) for 5 min.
*(2)* Analyze 0.1 mL aliquots of the supernatant solution as described in Figure **2020.07D**. In this assay, lactose is hydrolyzed to glucose plus galactose by β-galactosidase, sucrose is hydrolyzed to glucose and fructose by the sucrase enzyme (which has no action on FOS), and the remaining traces of maltose are hydrolyzed to glucose by maltase. Free galactose, glucose, and fructose are then determined.
- *Hydrolysis and measurement of isomaltose.—*
*(1)* Analyze 0.1 mL aliquots of the supernatant **[H(b)***(*2*)***]** as described in Figure **2020.07D**. To the bottom of a spectrophotometer cuvette, add 0.1 mL of sample solution plus 0.1 mL of solution **[D(f)]{**sucrase/maltase/β-galactosidase in 50 mM sodium maleate buffer (pH 6.5) plus BSA **[D(n)]}**. Also add 10 µL of oligo-1,6-β-glucosidase (1000 U/mL) suspension (Megazyme Cat. No. E-BGOG) and mix well. Incubate this solution at 30°C for 30 min.
*(2)* An alternative procedure involves adding 0.1 mL of 50 mM sodium maleate buffer (pH 6.5) plus BSA **[D(n)]** and 0.1 mL of sample solution to the spectrophotometer cuvette, and then add 10 µL of a suspension of sucrase (170 U/mL)/maltase (1000 U/mL)/β-galactosidase (1000 U/mL)/oligo-1,6-α-glucosidase (1000 U/mL) (Megazyme Cat. No. E-SMGG). Mix well and incubate at 30°C for 30 min.

**Figure 2020.07C.**
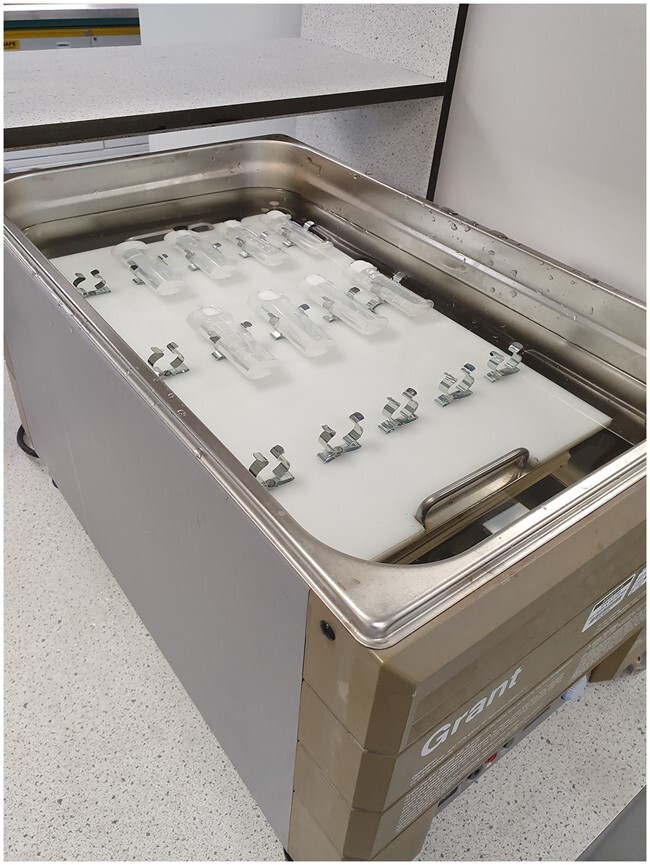
Polypropylene tube-holder in a Grant OLS 200 water bath to attach 40 mL polypropylene tubes containing samples.

**Figure 2020.07D.**
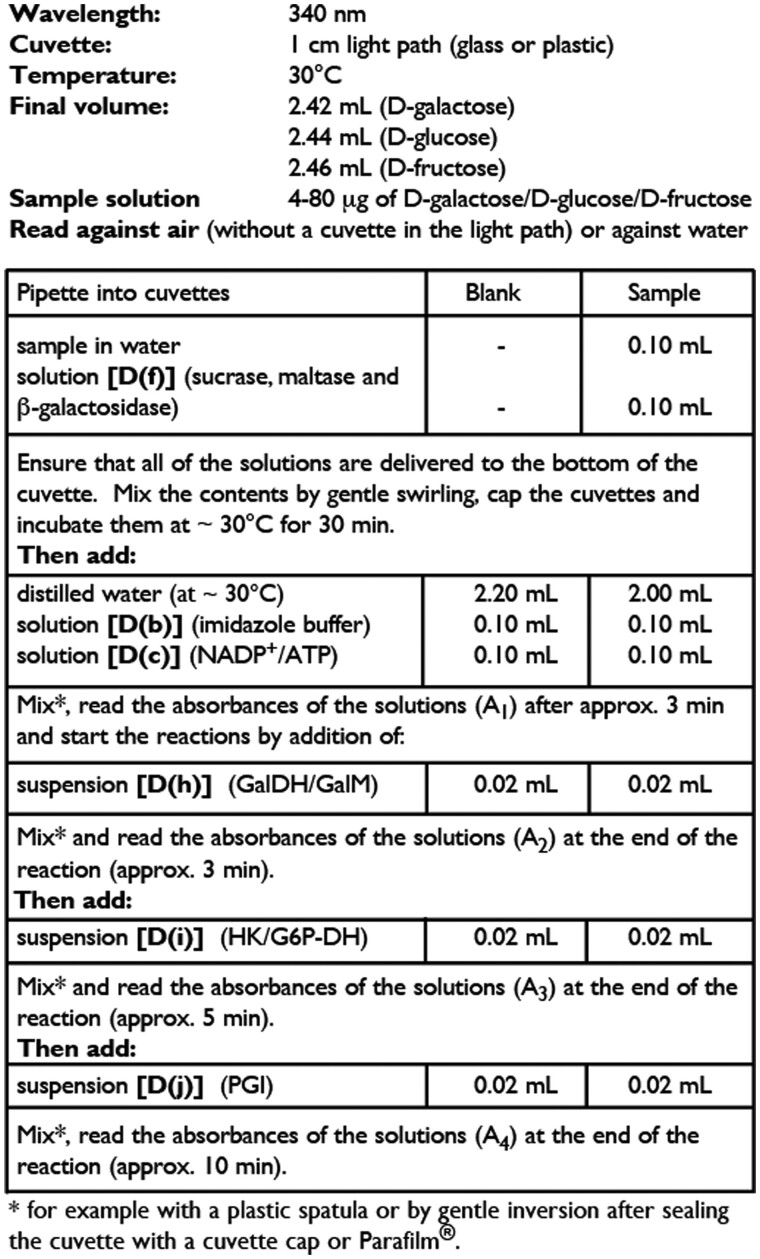
Procedure for the sequential measurement of galactose, glucose, and fructose in a spectrophotometer cuvette.

#### I. Calculations

Determine the absorbance difference (A_2_-A_1_) for both blank and sample. Subtract the absorbance difference of the blank from the absorbance difference of the sample, thereby obtaining ΔA_galactose_.

Determine the absorbance difference (A_3_-A_2_) for both blank and sample. Subtract the absorbance difference of the blank from the absorbance difference of the sample, thereby obtaining ΔA_glucose_.

Determine the absorbance difference (A_4_-A_3_) for both blank and sample. Subtract the absorbance difference of the blank from the absorbance difference of sample, thereby obtaining ΔA_fructose_.

The values of ΔA_galactose_, ΔA_glucose_, and ΔA_fructose_ should as a rule be at least 0.100 absorbance units to achieve sufficiently accurate results. If values are less than this, the possibility of decreasing the dilution of the sample extract or increasing the volume of the aliquot analyzed should be considered. This may not be possible if the combined absorbance then falls outside the analytical range of the assay. If the combined absorbance value is above 1.40, the sample should be diluted further and re-assayed.

The concentration of galactose, glucose and fructose can be calculated as follows:

$$c=\frac{V\;x\;\text{MW}\;x\;\Delta A\;x\;D}{\varepsilon \;x\;d\;x\;v}\;\;\;\;\;\left[g/L\right]$$

where V = final volume [mL]; MW = molecular weight of D-galactose, D-glucose or D-fructose [g/mol]; ε = extinction coefficient of NADPH at 340 nm = 6300 [l × mol^−1^ × cm^−1^]; d = light path [cm]; v = sample volume [mL]; and D= dilution factor (26-fold).

It follows for galactose:

$$\begin{array}{c} c=\frac{2.42\;x\;180.16\;x\;\Delta A_{\text{galactose}}x\;26}{6300\;x\;1.0\;x\;0.1}\;\;\left[g/L\right]\\ =17.993\;x\;\Delta A_{\text{galactose}}\;\;\;\;\;\left[g/L\right] \end{array}$$

for glucose:

$$\begin{array}{c} c=\frac{2.44\;x\;180.16\;x\;\Delta A_{\text{glucose}}x\;26}{6300\;x\;1.0\;x\;0.1}\;\left[g/L\right]\\ =18.142\;\;x\;\Delta A_{\text{glucose}} \end{array}$$

for fructose:

$$\begin{array}{c} c=\frac{2.46\;x\;180.16\;x\;\Delta A_{\text{fructose}}x\;26}{6300\;x\;1.0\;x\;0.1}\;\left[g/L\right]\\ =18.290\;x\;\Delta A_{\text{fructose}}\left[g/L\right] \end{array}$$

If oligo-1,6-α-glucosidase (10 µL) is added to the incubation mixture, appropriate adjustments of the final incubation volumes need to be made (i.e., for galactose, the final volume will be 2.43 mL, for glucose it will be 2.45 mL, and for fructose it will be 2.47 mL). When analyzing solid and semi-solid samples which are weighed out for sample preparation, the content (g/100 g) is calculated from the amount weighed as follows:

Content of galactose:

= c_galactose_ [g/L] x    EV  x 1  x 100 [g/100 g]

         1000 W

Content of glucose:

= c_glucose_ [g/L] x    EV  x 1  x 100 [g/100 g]

         1000 W

Content of fructose:

= c_fructose_ [g/L] x    EV x 1  x 100 [g/100 g]

         1000 W

where c_galactose_ [g/L] = concentration of galactose per L of undiluted extraction solution; c_glucose_ [g/L] = concentration of glucose per L of undiluted extraction solution; c_fructose_ [g/L] = concentration of fructose per L of undiluted extraction solution; EV = volume of solution used in the initial extraction (i.e., 20.5 mL); EV/1000 = adjustment from g/L of undiluted extraction solution to g/volume of extraction solution actually used; W = weight of sample analyzed in g;

Available carbohydrates (g/100 g)=galactose(g/100g)+glucose(g/100g)+fructose(g/100g)

*Note*: These calculations can be simplified by using the Megazyme *Mega-Calc*^TM^, downloadable from where the product appears on the Megazyme website (www.megazyme.com).

Measurement of galactose, glucose, and fructose using specific enzymes and following the increase in the reduction of NADP^+^ to NADPH is shown in Figure **2020.07E**.

**Figure 2020.07E.**
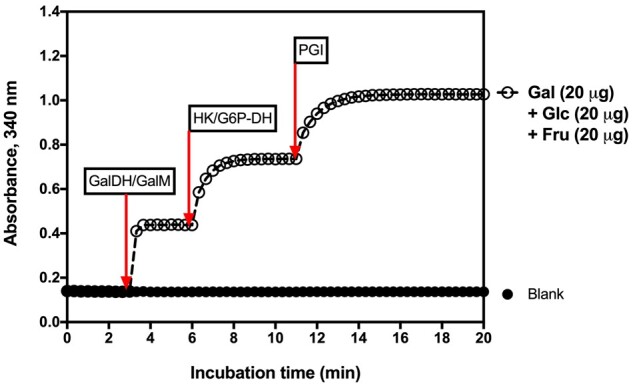
Increase in absorbance on incubation of a mixture of galactose, glucose and fructose with specific enzymes in the presence of NADP^+^, as detailed in Figure **2020.07D**.

#### J. Indicative Controls

Indicative controls are used as a check on assay conditions. Available carbohydrates, galactose, glucose, and fructose values for the included control sample should be within 3% of that stated on the control label. Inulin, levan, and galactosyl-sucrose oligosaccharides should not be hydrolyzed and thus should contribute nothing to the available carbohydrates value. Sucrose in FOS (e.g., Orafti P95^**^®^**^) will be hydrolyzed by the sucrase and, thus, correctly measured as available carbohydrates.

## Validation

### Planning

The purpose of this report is to verify and validate a method for measurement of available carbohydrates, defined as the sum of digestible starch, maltodextrins, maltose, sucrose, lactose, glucose, fructose, and galactose (and isomaltose if present). Digestible starch, maltodextrins, maltose, sucrose, lactose, and isomaltose are hydrolyzed to glucose plus fructose and/or galactose and the individual monosaccharides are then specifically measured. This method is an extension of AOAC Method **2017.16** (rapid integrated method for measurement of dietary fiber). Digestible starch and maltodextrins are hydrolyzed to glucose with PAA plus amyloglucosidase and sucrose is hydrolyzed to glucose plus fructose with a specific sucrase enzyme; lactose is hydrolyzed to galactose and glucose; the remaining traces of maltose are hydrolyzed by maltase to glucose and isomaltose is hydrolyzed to glucose.

*Performance characteristics*.—Performance characteristics that are investigated within this study are target selectivity (digestible starch and maltodextrins, maltose, isomaltose, sucrose, lactose, glucose, fructose, and galactose), specificity [no hydrolysis of oligosaccharides defined as dietary fiber, including inulin, FOS, levan, and galactosyl-sucrose oligosaccharides (e.g., raffinose)], recovery, LOD, LOQ, trueness (bias), and precision (repeatability).

*Working range*.—The assay follows the Megazyme Available Carbohydrates Assay Kit method (Megazyme Cat. No. K-AVCHO) and has a working range of 0.18–100% (w/w) available carbohydrates in the sample. For samples containing 0–10% (w/w) available carbohydrates, after incubation of 500 mg of sample with PAA/AMG in 20.5 mL of buffer, an aliquot (1.0 mL) is added to 5 mL of water (diluted 6-fold) and mixed, before centrifugation and analysis. For samples containing 10–100% (w/w) available carbohydrates, the same incubation conditions are performed, but 1 mL of the incubation mixture is added to 25 mL of water (diluted 26-fold) and mixed before centrifugation and analysis. The linearity of the reactions employed to measure galactose, glucose, and fructose are shown in Figure **2020.07A**. The r^2^ values were 0.9999 for each sugar. Also, the absorbance response with the three sugars is identical.

*Selectivity*.—The Available Carbohydrates Assay Kit is specific for digestible starch plus maltodextrins, maltose, isomaltose, sucrose, lactose, glucose, fructose, and galactose, as described. In the presence of highly purified PAA plus amyloglucosidase, digestible starch is hydrolyzed to glucose plus traces of maltose, and IMO are hydrolyzed mainly to glucose and isomaltose. Remaining traces of maltose are hydrolyzed to glucose with maltase; sucrose is quantitatively hydrolyzed to glucose and fructose by a specific sucrase enzyme which has no action on inulin, FOS, levan, or galactosyl-sucrose oligosaccharides (e.g., raffinose); lactose is quantitatively hydrolyzed to galactose and glucose and isomaltose is hydrolyzed to glucose.

**Figure 1.**
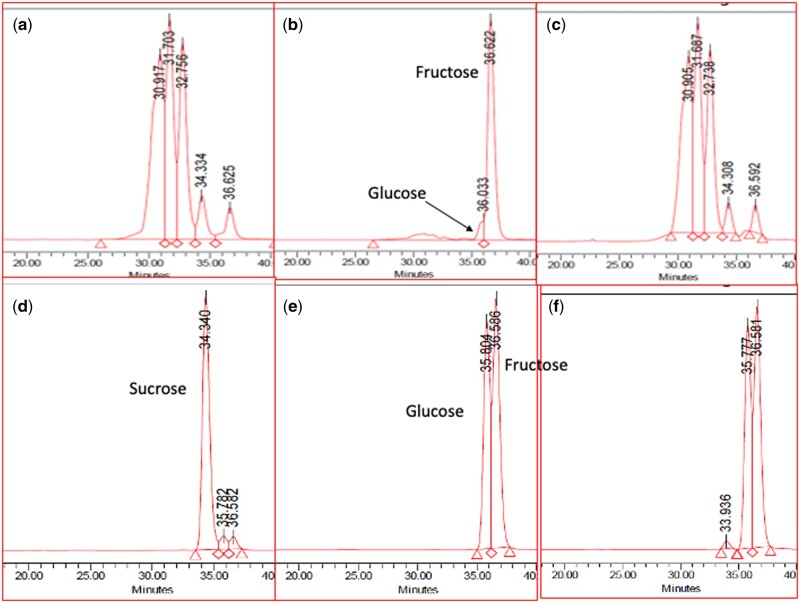
Hydrolysis of Orafti P95 (FOS) or sucrose by sucrase or invertase. Aliquots of Orafti P95 or sucrose solution (1 mL, 10 mg/mL) in water were incubated with 1.0 mL of sucrase (40 U on sucrose) in 25 mM sodium phosphate buffer (pH 6.5) or 1.0 mL of invertase (200 U) in 25 mM sodium acetate buffer (pH 4.5) at 40^°^C for various time intervals. Reactions were terminated by heating tubes at 100^o^C for 3 min and solutions were centrifuged at 13 000 rpm for 5 min. Solutions were analyzed by chromatography on TSKgel G2500PWXL^®^ HPLC columns with in-line deionization (a) Orafti P95 time zero; (b) Orafti P95 plus invertase for 10 min. (c) Orafti P95 plus sucrase for 60 min; (d) sucrose time zero; (e) sucrose plus invertase for 10 min; (f) sucrose plus sucrase for 10 min.

*Specificity*.—Levan, kestose, and raffinose are not hydrolyzed in the available carbohydrates assay procedure and thus do not add to the value determined. Commercial Orafti GR contains approximately 0.1% w/w fructose and 0.4% w/w sucrose. Orafti P95 preparation (FOS) contains approximately 5% w/w of fructose, glucose, and sucrose. Thus, an available carbohydrates value of approximately 5% w/w is obtained for Orafti P95 (Table 1). There is no hydrolysis of the FOS in this material. Confirmation of the lack of hydrolysis of Orafti P95**^®^**by sucrase, but rapid hydrolysis by invertase is shown in Figure 1. In these experiments, Orafti P95 or sucrose (10 mg) was incubated with either invertase (200 U, 1 mL, pH 4.5) or sucrase (40 U on sucrose, 1 mL at pH 6.5) at 40°C for various time intervals. Reactions were terminated by heating tubes in boiling water for 3 min and samples deionized and analyzed by HPLC on TSKgel^®^ G2500PW_XL_ columns, 30 cm × 7.8 mm, connected in series. Clearly, Orafti GR [Figure 1(a)] is rapidly hydrolyzed by invertase [Figure 1(b)] but not by sucrase [Figure 1(c)]. In contrast, sucrose [Figure 1(d)] is rapidly hydrolyzed by both invertase [Figure 1(e)] and sucrase [Figure 1(f)]. This experiment demonstrates both the effectiveness and specificity of sucrase, with completely hydrolysis of sucrose but no hydrolysis of FOS.

**Table 1.** Hydrolysis of Orafti P95, Orafti GR, levan, and raffinose in the available carbohydrates assay procedure

Sample	Free fructose and glucose in the sample^a^ , % w/w	Measured fructose and glucose after full AVCHO incubation, % w/w
Orafti P95 (FOS)	3.2	5.26^b^
Orafti GR	0.13	0.50
Levan	0.04	0.20
Raffinose	0.0	0.10

a In the Orafti P95 sample, 90% of the free sugar is fructose.

b The increase in the available carbohydrates value for Orafti P95 and Orafti GR is due to hydrolysis of sucrose in the sample.

Galacto-oligosaccharides (GOS) find use in the food industry as a prebiotic and as digestion resistant oligosaccharide mixtures. The β-galactosidase (MZ104) employed in the current method gives complete hydrolysis of lactose with limited hydrolysis of allolactose and GOS of DP 3 and greater (13) under the assay conditions used. Further knowledge on the likely degree of hydrolysis of commercial GOS preparations by β-galactosidase MZ104 as used in the method described here will provide information on likely overestimation of available carbohydrates in samples that are rich in these oligosaccharides.

**Figure 2.**
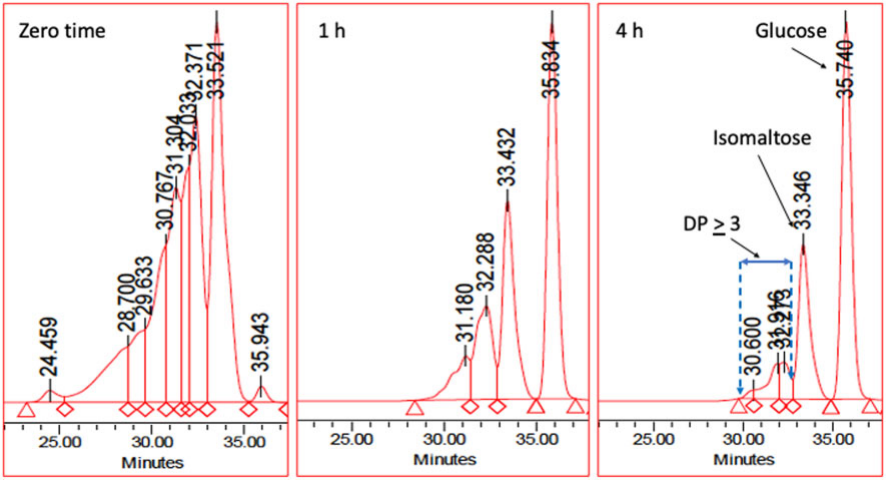
Hydrolysis of isomalto-oligosaccharides (e.g., Advantafiber) by PAA/AMG under the incubation conditions of the available carbohydrates procedure for 1 h and 4 h at 37^°^C. Samples of the incubation mixture were removed at 1 and 4 h and heated at 100^°^C to terminate the reaction. The solutions were desalted with resins in a tube and then analyzed by chromatography on TSKgel G2500PW_xL_ HPLC columns with in-line deionization.

**Figure 3.**
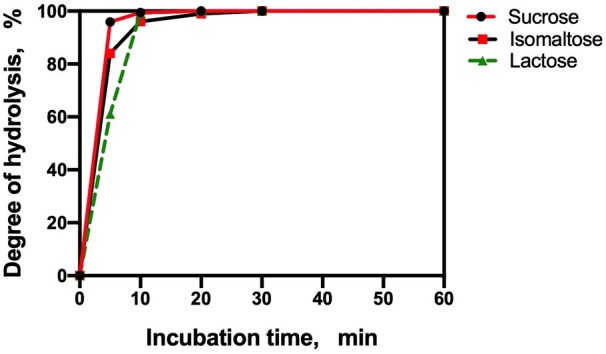
Hydrolysis of sucrose, lactose and isomaltose with 0.1 mL of the sucrase/maltase/β-galactosidase used in the available carbohydrates procedure **[D(f)]** plus 10 μL of oligo-1,6-α-glucosidase **[D(g)].**

Isomalto-oligosaccharides find application in food products as a source of “digestion-resistant” carbohydrate. The chromatographic patterns of a commercially available isomalto-oligosaccharide mixture, “Advantafiber**^®^**,” both before and after hydrolysis by PAA/AMG for 1 and 4 h, under the conditions of AOAC Method **2017.16**, are shown in Figure 2. After 4 h incubation with PAA/AMG, 60% of the oligosaccharides are converted to glucose, and 28% to isomaltose. Approximately 12% of the remaining oligosaccharides have a degree of polymerisation of ≥3 (i.e., only 12% can be defined as dietary fiber). This result is consistent with the ∼10% dietary fiber values obtained for this material using the Matsutani modification (AOAC Method **2001.03**) of AOAC Method **985.29** (McCleary, unpublished), and published data showing that isomaltose is hydrolysed by the mucosal α-glucosidases of the small intestine (7, 9, 10). The isomaltose (disaccharide) that is produced under the incubation conditions of AOAC Method **2017.16** is not defined as dietary fiber. Also, it is not measured as available carbohydrates because it is not hydrolyzed to glucose under the incubation conditions described in Figure **2020.07D**. Complete hydrolysis requires the use of another enzyme, namely, oligo-1,6-α-glucosidase to cleave the 1,6-α-glucosyl linkages in isomalto-oligosaccharides. Hydrolysis of sucrose, lactose, and also isomaltose can be achieved under the conditions defined in the available carbohydrates protocol (Figure **2020.07D**), if 10 µL of oligo-1,6-α-glucosidase (1000 U/mL) (Megazyme Cat. No. E-OAGUM) is included (Figure 3).

Oligo-1,6-α-glucosidase is now included in the enzyme mixture used to hydrolyze sucrose, maltose, and lactose provided in the Megazyme Available Carbohydrates test kit (K-AVCHO) The mixture contains sucrase (170 U/mL), maltase (1000 U/mL), MGZ104 β-galactosidase (500 U/mL), and oligo-1,6-α-glucosidase (1000 U/mL) as an ammonium sulphate suspension. In this case, sample extract in water (0.1 mL) and sodium maleate buffer (pH 6.5) containing BSA (0.1 mL) **[D(n)]** are added to the bottom of a spectrophotometer cuvette and the enzyme mixture (20 µL) is added and mixed and the solution incubated at 30°C for 30 min.

*Recovery*.—All values reported in this study are for the monosaccharide in the hydrated form. In starch, for example, glucose is in the anhydro form (MW = 162), thus hydrolysis of 100 g of starch yields ∼110 g of glucose. Presenting the carbohydrate as the weight of the particular monosaccharide simplifies subsequent calculations of the glycemic load of the particular food.

**Figure 4.**
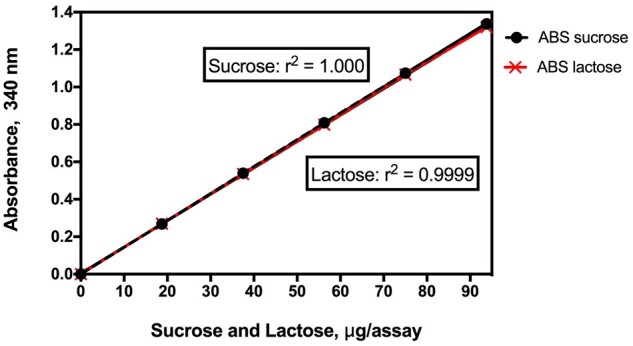
Recovery of sucrose and lactose (100–500 mg per incubation, i.e., 0–94 μg/assay)

For accurate measurement of available carbohydrates, the sucrase/β-galactosidase mixture must give complete hydrolysis of sucrose (with no hydrolysis of fructan or FOS; Figure 1) and of lactose. The quantitative hydrolysis and recovery of sucrose and lactose by the enzymes used in the available carbohydrates method is shown in Figure 4. Sucrose or lactose (500 mg) were dissolved in maleate incubation buffer (20.5 mL) and then aliquots (0.2–1.0 mL) were transferred to 25 mL of water and the volumes of each adjusted to 26 mL. Aliquots of this solution were incubated with sucrase/maltase/β-galactosidase as per Figure **2020.07D** and the amounts of glucose and fructose or glucose and galactose were measured spectrophotometrically, and the amounts of sucrose or lactose were determined (allowing for the molecular weight of these disaccharides). Results are shown in Figure 4 as sucrose and lactose. The recovery of sucrose or lactose across the five concentrations employed (equivalent to 100–500 mg/incubation) was 100%, demonstrating complete hydrolysis of these two disaccharides at the concentrations of the sucrase and β-galactosidase used in the assay.

In order to determine the effect of matrix on recovery of sucrose, lactose, or digestible starch, a variety of sample spiking experiments were performed. Six typical food samples were spiked with two levels of sucrose and lactose (5% w/w and 15% w/w) and of regular maize starch (10% w/w and 30% w/w), subjected to the complete incubation procedure and analyzed for galactose, glucose, and fructose. Concurrently, samples containing no spike were incubated and values obtained for galactose, glucose, and fructose obtained for these samples were subtracted from those obtained with the spiked sample. The data in Table 2 shows the recoveries obtained for galactose, glucose, and fructose were within 95–105% (w/w) for each of the samples studied, except for butter beans, in which case the recovery of the glucose component of both sucrose and lactose was ∼90% (w/w). The spiking experiments with butter beans and sucrose at 5% (w/w) were repeated numerous times and the recovery of the fructose was 98–104%, but the value for glucose ranged between 71 and 124%. It is considered that the high variation in recovery of the glucose component of the sucrose or lactose, but not the fructose or galactose of these added sugars, in butter beans samples is due to the fact that the glucose from the added sucrose or lactose is small compared to the glucose produced on hydrolysis of the starch in the sample. High absorbance values are obtained for the sample and the sample plus spike and the difference between the two absorbance values is small, leading to larger variations in the determined glucose value for the sucrose or lactose spike.

**Table 2.** Recovery^a^ of sucrose, lactose, and regular maize starch as glucose, fructose, and galactose from materials spiked with sucrose or lactose

Sample	Added sugar, g/100g	Recovered sugar, % w/w	
	Sucrose	Glucose	Fructose
Milled soybean (7.1 g AVCHO/100 g^b^)	5	98.6	98.4
	15	97.6	99.5
Broccoli (13.3 g AVCHO/100 g^b^)	5	100	95.5
	15	97.1	98.0
Chocolate peanuts (39 g AVCHO/100 g^b^)	5	102.8	103.0
	15	96.2	96.9
Cookies (61.7 g AVCHO/100 g^b^)	5	101.5	95.4
	15	95.2	99.0
All Bran Cereal (45 g AVCHO/100 g^b^)	5	102.9	104.6
	15	98.6	97.0
Canned butter beans (40 g AVCHO/100 g^b^)	5	66.7	95.5
	15	89.4	99.5
	
	**Lactose**	**Galactose**	**Glucose**
	
Milled soybean (7.1 g AVCHO/100 g)	5	102.9	104.4
	15	100.5	100.0
Broccoli (13.3 g AVCHO/100 g)	5	97.1	95.5
	15	103.1	100.0
Chocolate peanuts (39 g AVCHO/100 g)	5	104.4	97.1
	15	101.5	102.0
Cookies (61.7 g AVCHO/100 g)	5	100	98.7
	15	98.1	100.5
All Bran Cereal (45 g AVCHO/100 g)	5	97.1	100.0
	15	100.0	98.6
Canned butter beans (40 g AVCHO/100 g)	5	100.0	89.6
	15	98.1	92.6
	
	**Digestible starch**	**Glucose**	**—**
	
Milled soybean (7.1 g AVCHO/100 g)	10	100.0	—
	30	98.4	—
Broccoli (13.3 g AVCHO/100 g)	10	102.5	—
	30	98.3	—
Chocolate peanuts (39 g AVCHO/100 g)	10	98.4	—
	30	97.2	—
Cookies (61.7 g AVCHO/100 g)	10	97.6	—
	30	99.7	—
All Bran Cereal (45 g AVCHO/100 g)	10	107.2	—
	30	103.3	—
Canned butter beans (40 g AVCHO/100 g)	10	100.0	—
	30	100.6	—

a Recovery was determined by subtracting the available carbohydrates in the material from the available carbohydrates determined for the material containing the spike. The amount of sucrose added was determined as glucose and fructose after enzymatically hydrolyzing the sucrose to glucose and fructose. The recovered glucose was then determined as a percentage of that added as sucrose. The same procedure was used for fructose. In lactose and regular maize starch spiking experiments, the exact same determinations were made.

b The available carbohydrates value of the original material.

*LOD and LOQ*.—LOD (the lowest level at which detection of the analyte becomes problematic) and LOQ (the lowest level at which the performance of the assay is acceptably repeatable) of the available carbohydrates assay method were calculated by performing replicate assays (*n *=* *16) on a sucrose/cellulose sample with a relatively low content of available carbohydrates (5.17%, w/w). The analysis was performed as described except that the sample removed was added to 5 mL of water (instead of 25 mL) to obtain significant absorbance values in the assay. The LOD was 0.054% (w/w) using LOD = 3 × *s′_0_*, where *s′_0_* = the SD of replicate measurements. The LOQ was calculated as 0.179% (w/w) using LOQ = *k_Q_* × *s′_0_*, where *s′_0_* = the SD of replicate measurements. According to The International Union of Pure and Applied Chemistry, the default value for *k_Q_* = 10.

*Trueness (Bias)*.—Accuracy of the available carbohydrates assay method was assessed by comparison of the mean available carbohydrates content obtained for suitable reference materials with a specific reference value. There are no official CRMs for available carbohydrates, so the reference materials used for this analysis were a sucrose/cellulose mixture and a lactose/cellulose mixture.

Relative bias is calculated as follows:

$$b\left(\%\right)=\frac{\bar{x}\;-\;{\bar{x}}_{\mathrm{ref}}}{{\bar{x}}_{\mathrm{ref}}}\;\times 100$$

where *b*(%) = relative bias; x = mean available carbohydrates content; and x_ref_ = specific reference value.

The accuracy of the available carbohydrates assay method is extremely high with a calculated relative bias of 1.7% for the lactose/cellulose mixture and 3.4% for the sucrose/cellulose mixture.

*Precision*.—The precision of the available carbohydrates assay method was assessed using eight food samples. For each sample, duplicate extractions were processed and analyzed by the available carbohydrates assay on four separate occasions by a single analyst. The available carbohydrates content of the samples tested covered a working range of 44.1–88.9% (w/w) available carbohydrates. The repeatability (RSD_r_, %) across this sample set was extremely high (Table 3). The inter-day repeatability (RSD_r_, %) was less than or equal to 3.58% (w/w). This level of repeatability indicated a very high level of precision showing that the method is robust, reliable, and repeatable, and thus suitable for the application of measuring available carbohydrates in food and vegetable samples.

**Table 3.** Repeatability^a^ of the available carbohydrates assay procedure

	Available carbohydrates, % (w/w) dwb, mean ±2 SD, RSD_r_, %				
Sample	Day 1	Day 2	Day 3	Day 4	Inter-day mean, ±2 SD, RSD_r_, %
Wheat starch	87.3 ± 2.1	90.2 ± 2.2	88.2 ± 0.6	90 ± 1.1	88.9 ± 2.8
	1.21	1.21	0.34	0.61	1.60
All Bran	43.2 ± 2.2	45.4 ± 0.4	43.2 ± 1	44.5 ± 0.2	44.1 ± 2.2
	2.55	0.40	1.20	0.27	2.53
Sweet potato	59.6 ± 0.2	60.7 ± 1.3	58.2 ± 2.1	60.4 ± 1	59.7 ± 2.3
	0.14	1.10	1.80	0.81	1.92
Ripe banana	65.4 ± 0.4	70 ± 0.3	67.1 ± 1	66.8 ± 0.6	67.3 ± 3.6
	0.28	0.19	0.77	0.46	2.68
Carrot	53.7 ± 0.9	57.4 ± 0.6	55.1 ± 1.5	55.3 ± 0.3	55.4 ± 2.9
	0.87	0.54	1.36	0.23	2.58
Red pepper	51 ± 0.5	55.4 ± 2.5	53.8 ± 3.1	52.9 ± 1.9	53.2 ± 3.8
	0.49	2.25	2.92	1.83	3.58
Ryvita^®^	60.6 ± 1.3	61.5 ± 1.3	61 ± 2.4	62.6 ± 0.7	61.4 ± 2
	1.07	1.04	1.96	0.60	1.61
Swede	55 ± 4.3	53.6 ± 0.4	54.2 ± 1.3	54.1 ± 2	54.2 ± 2.1
	3.92	0.34	1.19	1.82	1.96

a The repeatability (RSD_r_, %) of the available carbohydrates assay method was assessed using eight milled samples. For each sample, duplicate extractions were processed and analyzed on 4 separate days. The available carbohydrate content of the samples tested covered a working range of 44.1–88.9% (w/w) on a dry weight basis. The repeatability (RSD_r_, %) across this sample data set was less than or equal to 3.58% for all samples.

The available carbohydrates method has been applied to the measurement of carbohydrates in a wide range of cereals and cereal based foods, dairy products, food, and vegetables and the results are shown in Table 4. Determinations were performed in duplicate.

**Table 4.** Available carbohydrate content^a^ of a range of cereal based foods, fruit, and vegetables and animal foods

Sample	Galactose, g/100 g^b^ “as is”	Glucose, g/100 g^b^ “as is”	Fructose, g/100 g^b^ “as is”	Available carbohydrates, g/100 g^b^ “as is”	Available carbohydrates, g/100 g^b^ “dwb”^c^
	
	Mean + 2 SD				
Cereal food products					
Weetabix	0 ± 0	66.3 ± 1.9	1.4 ± 0.1	67.7 ± 2	73.1 ± 2.2
Kelloggs All Bran	0 ± 0	36.1 ± 1.7	8.8 ± 0.6	44.9 ± 2.3	46.1 ± 2.4
Kelloggs Cornflakes	0 ± 0	80.0 ± 2.8	3.1 ± 0.2	83.1 ± 3.1	87.6 ± 3.2
Kelloggs Special K	0 ± 0.3	77.8 ± 0	6.1 ± 0.5	83.9 ± 0.2	87.5 ± 0.2
Kelloggs Frosties	0.1^d^ ± 0.2	70.1 ± 3.5	15.9 ± 0.1	86.0 ± 1.1	91.2 ± 1.2
Wholemeal Bread	0.1 ± 0	59.3 ± 0.3	0.1 ± 0.7	59.5 ± 0.4	65.7 ± 0.5
Ryvita^®^rye crackers	0 ± 0	59.9 ± 0.1	0 ± 0.1	59.9 ± 0.1	64.6 ± 0.1
Roma pasta	0 ± 0	74.2 ± 0.1	0 ± 0	74.2 ± 0.1	82.8 ± 0.1
Vegetables, fruits, and beans^e^					
Rooster potato	0 ± 0.1	33.6 ± 2.3	0.9 ± 0	34.5 ± 2.3	36.0 ± 2.4
Sweet potato	0.1 ± 0.1	41.8 ± 1.2	17.9 ± 0.4	59.8 ± 1.7	62.7 ± 1.8
Swede	0.2 ± 0.1	32.3 ± 1.1	20.1 ± 0.6	52.6 ± 1.6	55.2 ± 1.7
Carrots	0 ± 0	32.7 ± 1.5	26.7 ± 1.5	59.5 ± 3.1	60.8 ± 3.1
Red onion	0.3 ± 0.3	21.7 ± 1.3	19.2 ± 1.1	41.2 ± 2.0	46.7 ± 2.3
Broccoli	0.3 ± 0.5	11.3 ± 0.2	10.1 ± 0.5	21.8 ± 1.3	22.7 ± 1.3
Cauliflower	0.2 ± 0.1	16.2 ± 0.2	15.8 ± 0.2	32.2 ± 0.1	33.4 ± 0.1
Red kidney beans	0.1 ± 0	46.4 ± 0.1	0.5 ± 0.1	47 ± 0.2	48 ± 0.2
Butter beans	0 ± 0	43.9 ± 1.3	0 ± 2.4	44.4 ± 0	45.6 ± 0
Heinz baked beans	0.3 ± 0	41.1 ± 0.2	5.2 ± 0	46.6 ± 0.2	49.9 ± 0.2
Mushroom	0.1 ± 0	3.0 ± 0.2	0 ± 0	3.1 ± 0.2	3.4 ± 0.2
Ripe banana	0 ± 0.1	35.7 ± 1.7	30.6 ± 1.2	66.3 ± 3.0	72.4 ± 3.2
Mango (dried fruit pieces)	0 ± 0.1	35.3 ± 0.8	32.4 ± 0.8	67.7 ± 1.7	69.7 ± 1.7
Pineapple (dried fruit pieces)	0.1 ± 0.3	35 ± 2.4	34.1 ± 2.7	69.2 ± 4.9	79.0 ± 5.5
Goji berries (dried)	0 ± 0.2	25.6 ± 0	24.7 ± 0.2	50.4 ± 0.4	62.1 ± 0.5
Brussel sprouts	0.3 ± 0.3	19.7 ± 0.2	14.4 ± 0.3	34.3 ± 0.8	35 ± 0.9
Swede	0.1 ± 0.2	54.3 ± 1.5	7.5 ± 0.1	61.9 ± 1.8	63.7 ± 1.8
Butternut squash	1.4 ± 0.1	25.5 ± 0.5	25.2 ± 0.5	52.2 ± 1.2	54.2 ± 1.2
Chickpeas	0.1 ± 0.1	53 ± 1.1	0.5 ± 0.2	53.6 ± 0.8	54.3 ± 0.8
White cabbage	0.1 ± 0	23.4 ± 0.7	20.3 ± 0.5	43.7 ± 1.2	45.2 ± 1.2
Food and drink products					
Cow & Gate^®^ infant formula	29.4 ± 1.5	28.8 ± 1.6	0 ± 0	58.2 ± 3.0	59.4 ± 3.1
Angel Delight drink	1.7 ± 0.2	43.3 ± 0.8	24.9 ± 0.8	69.9 ± 1.5	70.9 ± 1.5
Birds Dream topping powder mixture	4.1 ± 0.3	16.7 ± 1.5	12.1 ± 1.4	32.9 ± 3.1	33.4 ± 3.2
Nescafe Gold Cappuccino^®^ powder	9 ± 0	46.6 ± 0	16 ± 0	71.6 ± 0	72.9 ± 0
Aero^®^ hot chocolate drink powder	6.2 ± 0.1	46.3 ± 0.2	20.9 ± 0.2	73.4 ± 0.5	74.6 ± 0.5
Nestle Nesquik^®^ drink powder	0 ± 0.1	43.2 ± 0.8	38.4 ± 0.6	81.6 ± 1.6	82.4 ± 1.6
Malted milk biscuits	0.4 ± 0.4	62.9 ± 0.9	7.0 ± 0.8	70.3 ± 2.1	72.4 ± 2.2
Chocolate biscuits	1.9 ± 0.3	53.1 ± 1.3	11 ± 0.3	66.0 ± 1.3	67.6 ± 1.4
Jam biscuits	0.1 ± 0	55.7 ± 0.7	12.7 ± 0.3	68.5 ± 0.9	71.8 ± 1.0
Shortbread finger biscuits	0.2 ± 0.1	57.5 ± 0.2	7.2 ± 0.2	65 ± 0.6	66.8 ± 0.6
Cadbury^®^ Dairy Milk chocolate	3.7 ± 0.3	26.8 ± 0.2	22.1 ± 0.2	52.6 ± 0.7	53.3 ± 0.7
Animal feeds and foods					
Red Mills^®^ hen pellets	0 ± 0.1	41.9 ± 0.8	1.2 ± 0.3	43.1 ± 0.6	47.3 ± 0.7
Gain^®^ soybean meal	0.2 ± 0.3	6.2 ± 0	3.1 ± 0.3	9.4 ± 0	10.5 ± 0
Gain^®^ low starch horse feed	0 ± 0.1	11.2 ± 0.6	2.3 ± 0	13.5 ± 0.7	15.1 ± 0.8
Gain ^®^Drive dairy feed complete	1.1 ± 0.1	21.2 ± 0.1	1.0 ± 0.2	23.3 ± 0.2	26.9 ± 0.2
Brett Brothers^®^ animal feed	0 ± 0.1	44.2 ± 0.5	0.7 ± 0.1	45 ± 0.3	51.6 ± 0.4
Adult kibble dog food	0 ± 0	43.4 ± 0.4	0 ± 0	43.4 ± 0.4	46.1 ± 0.4
Purina GoCat^®^ cat food	0 ± 0.1	35.9 ± 0.1	1.7 ± 0.3	37.7 ± 0.3	40.2 ± 0.3
Gain^®^ feeds flaked maize	0 ± 0.1	71.9 ± 3.3	0 ± 0	71.9 ± 3.2	82.9 ± 3.6
Gain^®^ premium cut wet dog food	0 ± 0	0.6 ± 0.1	0 ± 0	0.6 ± 0.1	0.6 ± 0.1
Native and modified starches					
Regular maize starch, Megazyme lot 60401	0 ± 0	91.9 ± 1.9	0 ± 0	91.9 ± 1.9	103.4 ± 2.1
Hylon VII^®^ (high amylose starch)	0 ± 0	39.7 ± 0.9	0 ± 0	39.7 ± 0.9	45.2 ± 1.1
Wheat starch, Sigma S-5127	0.1 ± 0	92.3 ± 4.4	0 ± 0	92.4 ± 4.2	103.8 ± 4.8
Rice starch, Sigma S-7260	0.1 ± 0	90.4 ± 3.4	0 ± 0	90.5 ± 3.4	101.8 ± 3.8
High amylopectin Corn starch, Sigma S-9679	0.1 ± 0	85.4 ± 0.8	0 ± 0	85.5 ± 0.8	96.1 ± 0.9
Corn starch, Sigma S-4126	0 ± 0.2	91.7 ± 7.1	0 ± 0	91.7 ± 7.2	103.8 ± 8.2
Barley flour, Megazyme 60301	0.1 ± 0.1	69 ± 0.2	0 ± 0	69.1 ± 0.1	77.8 ± 0.1
Barley Max^®^ (The Healthy Grain Pty. Ltd)	0.1 ± 0	32.2 ± 0.5	5.0 ± 0.1	37.3 ± 0.6	40.2 ± 0.7
Potato starch, Sigma S-4251	0 ± 0.2	49.5 ± 0.4	0 ± 0	49.5 ± 0.5	57.7 ± 0.6
Potato Amylose, Sigma A-9262	0.2 ± 0.1	57.6 ± 0.5	0 ± 0	57.8 ± 0.6	64.7 ± 0.7
Fibersol 2, Matsutani Chemical Co.	0.2 ± 0.1	13.6 ± 0.3	0 ± 0.1	13.8 ± 0.4	14.9 ± 0.5
Fibersym, MGB Ingredients	0.1 ± 0	34.6 ± 0.3	0 ± 0	34.7 ± 0.3	38.9 ± 0.4

a All analyses were performed in duplicate.

b Values presented are as g (of the particular monosaccharide)/100 g of material. Values have not been corrected for the water of hydration of the sugars in the particular disaccharide or polysaccharide.

c Dry weight basis.

d Under the assay conditions employed, a galactose value of 0.3% (w/w) equates to an absorbance difference of 0.004. Absorbance value differences below 0.005 are insignificant. For more accurate estimation of galactose in such materials, assays should be performed on the original incubation mixtures (i.e., 20.5 mL) without dilution.

e Vegetable, fruit, and wet bean materials were freeze-dried before analysis. The “as is” values for these materials refer to the freeze-dried powders. Moisture content was determined for all materials to allow calculation of “available carbohydrates” on a dry weight basis.

*Stability*.—The Available Carbohydrates Assay Kit as formulated by Megazyme comes with a two-year stability guarantee from date of purchase. When dissolved, the PAA pancreatic α-amylase/amyloglucosidase **[D(d)]** must be stored on ice and used on the day of preparation. However, if prepared as an ammonium sulphate suspension, it is stable at 4°C for 3 months. All other kit components, if stored as recommended in the kit booklet, are stable for more than 2 years. Individual kit components may have longer stability guarantees: this information is available on the component label as the expiry date. Regular quality control testing is performed within the Megazyme QC laboratory.

## Discussion

Available carbohydrates have been defined (1, 2) as the sum of sugars (glucose, fructose, galactose, sucrose, maltose, and lactose) and complex carbohydrates (“malto” dextrins, starch, and glycogen). Historically, these have been measured individually by a combination of enzymatic, complex chemical (2), and HPLC procedures and the values pooled. The method described here involves the complete and specific hydrolysis of each carbohydrate to the component monosaccharides, glucose, fructose, and galactose and specific enzymatic measurement of these in a single reaction cuvette. With the increased knowledge of starch hydrolysis in the human small intestine and the recognition of the importance of resistant starch as a component of dietary fiber, it has become important to specifically measure digestible starch rather than total starch for the calculation of available carbohydrates. Consequently, food digestion in the small intestine has been simulated using a combination of gentle shaking or stirring in the presence of PAA/AMG at 37°C for 4 h, as is employed in AOAC Method **2017.16** (7, 8). A sample of the incubation solution is removed, diluted in water, and an aliquot centrifuged. This solution is used in the determination of available carbohydrates. In the incubation with PAA and AMG, maltose, maltodextrins, glycogen, and digestible starch are hydrolyzed to D-glucose (with trace levels of maltose). On subsequent incubation with β-galactosidase (13), sucrase, and maltase, lactose is hydrolyzed to glucose and galactose, sucrose is specifically hydrolyzed to glucose and fructose and remaining traces of maltose are hydrolyzed to glucose (Figure **2020.07D**). Incubation with oligo-1,6-α-glucosidase gives complete hydrolysis of isomaltose (derived from IMO) to glucose. Galactose, glucose, and fructose are then measured enzymatically, as shown in Figure **2020.07E**. Traditionally, sucrose has been hydrolyzed using invertase (β-fructofuranosidase), but this enzyme also acts on FOS resulting in overestimation of the sucrose (Figure 1). Clearly, invertase is not suitable for the specific hydrolysis of sucrose in the presence of FOS The β-galactosidase employed here is MZ104 β-galactosidase (Megazyme) which has optimal activity at pH 6.5 which makes it ideal for use in conjunction with sucrase which has a similar pH optimum.

The available carbohydrates content of a number of food materials is shown in Table 4. The repeatability of the available carbohydrates method has been determined by analyzing eight food materials in duplicate over 4 days (Table 3). Inter-day repeatability is excellent, with RSD_r_ values ranging from 1.6 to 3.58.

The currently described “available carbohydrates” method together with the total dietary fiber method (AOAC Method **2017.16**) (8) allows the measurement of all carbohydrates, including digestible and resistant starch, in food materials. For food labeling in the United States, the only approved method for labeling of carbohydrates in the nutrition facts panel involves subtracting the amount of crude protein, total fat, moisture, and ash from the total sample weight. Dietary fiber is included in the total carbohydrate value, but it can also be reported separately on the label. “Net carbohydrates” is determined by subtracting the dietary fiber value from the total carbohydrates value. A major problem in using this approach is that of accumulated errors (4). In this paper, a simple method is described for the direct measurement of the available carbohydrates (glucose, fructose, galactose, sucrose, lactose, isomaltose, maltodextrins, and digestible starch) by direct measurement of glucose, fructose, and galactose. Such values also allow estimation of the glycemic index value of a food.

## Conclusion

The method outlined in this document is a robust, quick, and easy method for the direct measurement of digestible carbohydrates in a broad range of human and animal foods and feeds. Data presented in this report verifies and validates that this method is fit for the purpose intended.
